# A Study on the Efficiency of Tourism Poverty Alleviation in Ethnic Regions Based on the Staged DEA Model

**DOI:** 10.3389/fpsyg.2021.642966

**Published:** 2021-04-12

**Authors:** Jianchun Yang, Ying Wu, Jialian Wang, Chengcheng Wan, Qian Wu

**Affiliations:** School of Business Administration, Guizhou University of Finance and Economics, Guiyang, China

**Keywords:** ethnic regions, tourism poverty alleviation efficiency, two-staged Data Envelopment Analysis, Malmquist index, tourism

## Abstract

Poverty alleviation through tourism is an important way for China to achieve targeted poverty alleviation and win the battle of poverty alleviation. As a region with deep poverty and great difficulty in poverty alleviation, whether tourism development has injected key impetus into ethnic minority areas needs to be tested by both qualitative analysis and quantitative measurement. This paper takes eight ethnic provinces (regions) in China as an example to conduct an empirical study. Based on the Data Envelopment Analysis (DEA)-BCC model and Malmquist index, it evaluates the tourism investment and tourism poverty alleviation efficiency of the ethnic regions in the two stages of tourism poverty alleviation, and analyzes them by classification. The results of the study show: (1) The pure technical efficiency in the first stage is relatively high, but the total factor productivity of each region is declining; (2) The pure technical efficiency in the second stage is also relatively high, but the scale efficiency is low, and the change rate of total factor productivity of the provinces in China has increased significantly; (3) The “double high” type includes Guangxi, Inner Mongolia, and Guizhou, and the “double low” type includes Qinghai, Yunnan, Tibet, Xinjiang, and Ningxia. The results of the study generally show that tourism poverty alleviation has brought about the improvement of the living standards of residents and the development of local economy, but the efficiency of tourism poverty alleviation needs to be improved. On this basis, the article puts forward corresponding improvement measures, in order to further help the ethnic minority areas get rid of poverty in a comprehensive way by promoting the efficient and sustainable development of tourism.

## Introduction

Poverty is one of the major social problems facing humankind, a severe test that China faces in building a moderately prosperous society in an all-round way, and the focus of attention for all sectors of society in order to achieve social stability, enhance people's well-being, and promote human development and progress (Davidson and Sahli, 2015). Practice has proved that poverty alleviation by tourism has become an important way for poverty-stricken areas to escape the poverty trap and is a regional development model that drives poor areas with better tourism resources to develop their economy and achieve prosperity (Medina Muñoz and Gutiérrez Pérez, 2016). The tourism poverty alleviation of China began to rise at the end of the last century. In recent years, the slogan of “targeted poverty alleviation” has been put forward and further refined, and the development of tourism industry in poverty-stricken areas has been promoted through the development of characteristic cultural tourism. From 2010 to 2014, more than 10% of the poor people were lifted out of poverty through the development of tourism across the country, and more than 10 million people were lifted out of poverty through tourism (He and Wang, 2019). Winning the battle against poverty is the bottom line task of building a moderately prosperous society in an all-round way as scheduled in 2020. As of May 16, 2020, there are still 52 state-level poverty-stricken counties in China that have not yet been lifted out of poverty. They are distributed in seven provincial administrative regions in China, including Xinjiang Uygur Autonomous Region, Yunnan Province, Guizhou Province, Guangxi Zhuang Autonomous Region, and Ningxia Hui Autonomous Region. The five provincial-level administrative regions all belong to the eight ethnic provinces (hereinafter referred to as ethnic regions), and the number of impoverished counties in ethnic regions accounts for more than 71% of the total. It can be seen that ethnic regions are currently the top priority in China's overall victory in the battle against poverty. Due to the relatively backward socioeconomic development level, inconvenient transportation, and other factors in the ethnic areas, their natural landscapes, ethnic customs, and cultural customs are relatively intact, their tourism resources are well-endowed, and tourism is developing rapidly. Tourism poverty alleviation is one of the important measures to achieve targeted poverty alleviation in China. However, whether the development of tourism can effectively alleviate poverty in ethnic areas is still doubtful. It is urgent to quantitatively measure and evaluate the efficiency of tourism poverty alleviation in ethnic areas in order to improve tourism in ethnic areas. The efficiency of poverty alleviation promotes the sustainable development of poverty alleviation by tourism in ethnic areas (Yang et al., 2020).

Tourism poverty alleviation is a special development approach; there is a good synergy and coupling relationship between tourism and poverty alleviation development (Croes and Vanegas, 2008; Zhang, 2019; Wang et al., 2020a), which has an important impact on driving the economic development of poor regions and lifting the poor out of poverty (Kim et al., 2016; Wang et al., 2020). Since the 1980s, tourism poverty alleviation as an effective anti-poverty measure has attracted the attention of scholars at home and abroad. After Ashley proposed “Tourism for the Poor People” (PPT) (Ashley et al., 2001) and Sofield et al. proposed the concept of “Sustainable Tourism for Poverty Elimination” (ST-EP) (Sofield et al., 2003), poverty alleviation by tourism has now become an important research content of the domestic and foreign tourism industry and academic circles (Zhang and Zhang, 2005). Qin and others explored the relationship between tourism poverty alleviation and the local ecological environment (Qin et al., 2020), Rogerson studied the effect of tourism poverty alleviation on the economic development of South Africa (Rogerson, 2006), Chok et al. discussed the impact of tourism poverty alleviation on sustainable development (Chok et al., 2007), and Hall focused on exploring the impact of tourism poverty alleviation on the development of southern countries' benefit (Hall, 2007). In recent years, academic research on tourism poverty alleviation has gradually extended from the concept, development model (Jin et al., 2019), influencing factors (Lv et al., 2020), and implementation path of tourism poverty alleviation (Guo, 2020) to the investigation of the efficiency of tourism poverty alleviation (Liang et al., 2020). Through a review of the extensive literature, existing relevant studies have focused on measurement methods, tourism poverty alleviation regions, temporal evolution, industry sectors, and efficiency improvement measures; among them, improving tourism development capacity is an important measure (Dias et al., 2020). Some scholars have found that the resource allocation and management level of tourism poverty alleviation in ethnic areas has been greatly improved, and the pure technical efficiency is relatively high, but the distribution of tourism resources still needs to be further optimized (Zapata et al., 2011; Chen and Wang, 2020).

Most of the domestic and international scholars use Data Envelopment Analysis (DEA) for efficiency evaluation (Habibov and Fan, 2010; Xu and Chen, 2015), and have explored the combination of traditional DEA with other methods, such as with the Malmquist index (Chen et al., 2018; Lu et al., 2019; Yin and Tan, 2019), the two-stage bootstrap-DEA method (Ren et al., 2016; Chaabouni, 2019), the three-stage DEA method (Cao and Ma, 2017; Zhao H. et al., 2018), the Super-SBM model (Croes and Rivera, 2017; Wang et al., 2019), and the DEA-Tobit method (Heyuan and Xiaoling, 2016). Others have measured efficiency through the use of AHP hierarchical analysis (Zhang and Xiang, 2016) and gray correlation analysis (Deng et al., 2015). The traditional DEA method is the most commonly used method to evaluate the efficiency of tourism poverty alleviation (Han et al., 2019; Wang and Li, 2019), but it easily leads to deviations in the measured tourism poverty alleviation efficiency value, which needs to be further optimized. The existing literature mainly covers the study area including physical and geographical regions such as the western region (Feng et al., 2020) and the Wuling Mountains (Long et al., 2015; Wang et al., 2020b), as well as contiguous areas of special hardship such as the 20 poor counties (cities and districts) in the Wuling Mountains Hunan area (Huang, 2017) and Dabie Mountains (Liang et al., 2020). In addition, it also covers minority regions in Hainan Province (Yan et al., 2018), Xiangxi Tujia and Miaoze Autonomous Prefecture (Zhang and Xiang, 2016), 12 allied cities in Inner Mongolia (Wu and Liu, 2018), and Enshi Tujia and Miao Autonomous Prefecture (Wang and Lin, 2020). As far as the research objects are concerned, most of them are targeted at the country as a whole, individual provinces or geographically concentrated poverty-stricken regions, lacking a targeted comparative study of the efficiency of tourism poverty alleviation in various ethnic provinces (regions), weakening the reference value of improving poverty alleviation in poor ethnic regions. In terms of research content, scholars have used a variety of research methods to evaluate the efficiency of tourism poverty alleviation in multiple regions and industry sectors (Xu and Chen, 2015; Wang and Guo, 2018), and some scholars have conducted static and dynamic analysis and evaluation in conjunction with the Malmquist index to identify the main reasons affecting the change in total factor productivity of tourism poverty alleviation (Dias et al., 2020). Other scholars have studied the spatial differentiation of the efficiency of tourism poverty alleviation by combining the spatial analysis function of GIS, analyzing the global spatial autocorrelation of the overall efficiency of tourism poverty alleviation (Deng and Zhang, 2017), revealing the aggregation benefits and inter-regional gaps that tourism poverty alleviation possesses in space (Yang et al., 2018), and proposing corresponding measures to improve the efficiency of tourism poverty alleviation (Sun and Zhang, 2018). The efficiency of tourism poverty alleviation consists of two stages: the evaluation of the efficiency of tourism development and the evaluation of the efficiency of tourism output for poverty alleviation, but most of the existing literature only studies the efficiency of the output of tourism poverty alleviation without considering the efficiency of the input stage of tourism poverty alleviation, resulting in the inability to more objectively evaluate the overall efficiency of poverty alleviation and trace the more essential influencing factors of tourism poverty alleviation.

In general, in sharp contrast to the practice of tourism poverty alleviation, relevant research on the efficiency of tourism poverty alleviation in ethnic areas is still lagging behind, especially on the input–output efficiency of tourism poverty alleviation in ethnic regions, and the relevant results are also scattered, so there is an urgent need to verify the promotion effect of relevant tourism poverty alleviation policies in greater depth, so as to scientifically propose future development strategies and development paths for tourism poverty alleviation in ethnic regions. Therefore, this paper adopts the two-stage DEA-BCC model combined with Malmquist index to conduct empirical research, using eight provinces (regions) of China's ethnic groups as case sites to study the efficiency of tourism investment and tourism poverty alleviation in two stages for quantitative measurement and evaluation, to analyze the factors influencing the efficiency of tourism poverty alleviation, and to propose effective countermeasures to promote the efficiency of tourism poverty alleviation. Through more effective research methods and a more complete research process, in order to obtain more accurate and more valuable research results, it has important theoretical and practical significance for the positioning of tourism poverty alleviation in ethnic areas, the enhancement of the efficiency of tourism poverty alleviation, and the realization of comprehensive and precise poverty alleviation strategic planning, and provides reference for the development of poverty alleviation work in poor ethnic regions in China.

## Research Design

### Research Methodology

#### Data Envelopment Analysis Analysis Method

Data Envelopment Analysis analysis method, also known as DEA method, is mainly used to evaluate the efficiency of complex systems with multiple inputs and multiple outputs. The basic models include the CCR model under the assumption of constant returns to scale and the BCC model under the assumption of variable returns to scale. The biggest advantage of the BCC model is that it eliminates the assumption of constant returns to scale. Using this model, the technical effectiveness and scale effectiveness of the decision-making unit can be evaluated. Compared with the CCR model, it can provide more effective management information and evaluation results (Zhao C. et al., 2018). This paper selects the BCC model to evaluate the efficiency of tourism poverty alleviation. The results of DEA include comprehensive efficiency, technical efficiency, scale efficiency, and scale returns. Comprehensive efficiency reflects the allocation, utilization, and scale agglomeration efficiency of element resources. Technical efficiency reflects the allocation and utilization efficiency of factor resources. Scale efficiency reflects the scale agglomeration efficiency of factor resources. Returns to scale are divided into three situations: increasing returns to scale (IRS), constant returns to scale (CRS), and diminishing returns to scale (DRS).

Suppose there are n DMUs representing input and output. Each DMU corresponds to m types of inputs and s types of outputs, which are represented by vectors *X*_*ij*_ and *Y*_*rj*_, namely:

$$\begin{array}{l} \begin{array}{c} X_{ij}={\left(x_{1j},x_{2j},...,x_{ij}\right)}^{T},j=1,2,...,n\\ Y_{rj}={\left(y_{1j},y_{2j},...,y_{rj}\right)}^{T},j=1,2,...,n \end{array} \end{array}$$

Among them, *X*_*ij*_ is the *i* type of input of any decision-making unit DMU, and *Y*_*rj*_ is the *r* type of output of any decision-making unit DMU. For each DMU, the investment-oriented BCC model is as follows:

$$\begin{array}{l} \text{s}.t.\left\{\begin{array}{l} \min\theta =\theta_{0}\\ \Sigma_{j=1}^{\text{n}}\lambda_{j}y_{j}-s^{+}=y_{0}\\ \Sigma_{j=1}^{\text{n}}\lambda_{j}x_{j}+s^{-}=\theta x_{0}\\ \Sigma_{j=1}^{n}\lambda_{j}=1\\ \lambda \geq 0j=1,2,...n;s^{+}\geq 0;s^{-}\geq 0 \end{array}\right\} \end{array}$$

When *θ* = *1 and s*^−^ = *s*^+^ = *0*, the decision unit DEA is judged to be valid, indicating that the input–output of tourism poverty alleviation in the region happens to reach the optimal efficiency; when *θ* < *1 and s*^−^*or s*^+^ ≠ *0*, the decision unit is judged to be DEA invalid, indicating that the input–output of tourism poverty alleviation in the region has not reached the optimal efficiency.

#### Malmquist Index Model

The Malmquist index was first proposed by Malmquist (1953) and later improved and defined by Caves et al. (1982). The Malmquist index is an efficiency evaluation method based on a distance function^1^ that dynamically reflects the change and trend of the relative efficiency of the research object (Zhang et al., 2013). The Malmquist model is based on DEA and calculates the input–output efficiency through the distance function ratio, which can reflect the dynamic changes in relative efficiency from multiple angles and levels. The model is constructed as follows:

According to the definition of the Malmquist TPF index by Caves et al. (1982), the Malmquist TPF index based on the technology of *t* period is:

$$\begin{array}{l} M^{\text{t}}=\frac{D^{t}\left(x^{t},y^{t}\right)}{D^{t}\left(x^{t+1},y^{t+1}\right)} \end{array}$$

The same as above, the Malmquist TPF index based on technology in *t* + 1 period is:

$$\begin{array}{l} M^{t+1}=\frac{D^{t+1}\left(x^{t},y^{t}\right)}{D^{t+1}\left(x^{t+1},y^{t+1}\right)} \end{array}$$

In order to avoid the arbitrariness in the selection of production technology reference, the geometric mean of the two Malmquist TFP indices is used as the measurement of the input-oriented Malmquist TFP, which is:

$$\begin{array}{l} \begin{array}{c} M\left(x^{t+1},y^{t+1},x^{t},y^{t}\right)={\left[\left(\frac{D^{t+1}\left(x^{t},y^{t}\right)}{D^{t+1}\left(x^{t+1},y^{t+1}\right)}\right)\left(\frac{x-\mu }{\sigma }\right)\right]}^{\frac{1}{2}}\\ =\frac{D^{t}\left(x^{t},y^{t}\right)}{D^{t+1}\left(x^{t+1},y^{t+1}\right)}\left(\frac{D^{t+1}\left(x^{t+1},y^{t+1}\right)}{D^{t}\left(x^{t+1},y^{t+1}\right)}\frac{D^{t+1}\left(x^{t},y^{t}\right)}{D^{t}\left(x^{t},y^{t}\right)}\right) \end{array} \end{array}$$

If the value of the Malmquist TPF index is >1, it indicates that the total factor productivity level of the decision-making unit *t* + 1 period has increased compared with the previous period. If the value of the Malmquist TFP index is equal to 1, it indicates that the total factor productivity between the two periods is consistent. If the value of the Malmquist TPF index is <1, it means that total factor productivity has fallen.

### Evaluation Index

#### Sample Selection and Data Sources

Considering the representativeness and typicality of sample selection, as well as improving the richness of tourism poverty alleviation research objects, this paper selects ethnic regions in China with more backward economic development, deeper poverty, and better tourism resource endowment to study the efficiency of tourism poverty alleviation, including the eight provinces of Guangxi Zhuang Autonomous Region, Inner Mongolia Autonomous Region, Guizhou Province, Yunnan Province, Tibet Autonomous Region, Qinghai Province, Ningxia Hui Autonomous Region, and Xinjiang Uygur Autonomous Region. Based on the stage of vigorous development of China's tourism poverty alleviation, the selection of the study period by relevant scholars, and the availability of data, this paper selects input–output indicators for ethnic regions of China from 2003 to 2017, and the following data are obtained from the China Statistical Yearbook, China Tourism Yearbook, and provincial statistical yearbooks.

#### Selection of Indicators

**Figure 1:**
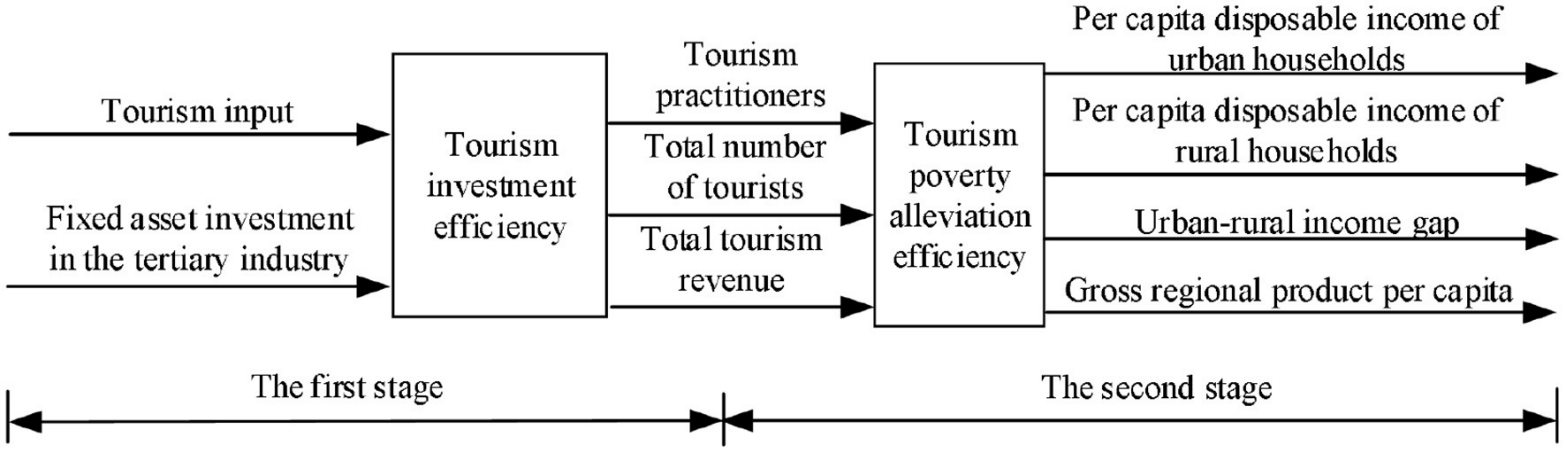
Two-stage model.

The process of tourism poverty alleviation should include the process from the input of tourism development elements to the output of tourism development level and then to the output of tourism poverty alleviation benefits, that is, the process of tourism input and output and the transformation of tourism output into tourism poverty alleviation benefits. Therefore, the evaluation of the efficiency of tourism poverty alleviation in this paper will also proceed from two stages, namely, the evaluation of tourism development efficiency and the efficiency evaluation of tourism output of poverty alleviation benefits. This is shown in Figure 1.

The first stage is the evaluation of tourism development efficiency. By consulting a large number of relevant literature, this paper selects two variables, “tourism investment” and “fixed asset investment in the tertiary industry,” as the input variables of tourism industry. “Tourism investment” refers to the financial support provided by the state for the development of tourism in various regions. “Fixed asset investment in the tertiary industry” refers to the amount of fixed asset investment in the tertiary industry, including transportation, accommodation, and catering. At present, most of the output indicators of tourism development are based on the two indicators “total tourism revenue” and “total number of tourists.” The “total tourism revenue” refers to the sum of domestic tourism revenue and inbound tourism revenue, representing the economic output of the tourism industry. The “total number of tourists” is the sum of the number of domestic tourist receptions and the number of inbound tourists, which represents the scale of tourism industry output. This paper adds “tourism employees” as an output variable of the tourism industry on the basis of these two indicators because the employment of local poor residents driven by the development of tourism can be seen as one of the contributions of tourism to poverty alleviation.

The second stage is the evaluation of the poverty alleviation efficiency of tourism output. The tourism output indicators in the first stage are the input indicators in the second stage. In order to more accurately measure the poverty alleviation efficiency of tourism output, it is necessary to separate the poverty alleviation benefits generated by the tourism industry from the data jointly produced by various sectors of the national economy. At present, some scholars regard the ratio of total tourism revenue to GDP as the economic contribution of tourism, and use the idea of equal substitution to replace the proportion of per capita disposable income from tourism with the contribution of tourism, so that we can calculate the contribution of tourism in per capita disposable income. This paper calculates the per capita disposable income tourism contribution of rural residents and the disposable income tourism contribution of urban residents in each province to express the economic benefits generated by tourism. The “urban–rural income gap” is measured by the urban–rural income gap index, which refers to the ratio of the disposable income of urban residents to the per capita net income of rural residents. The “gross regional product per capita” refers to the market value of all final products (goods and services) produced by the region's economy and society using the factors of production within a certain period of time, and is an important indicator of a region's economic situation.

## Empirical Analysis

### Static Efficiency Analysis Based on Variable Returns to Scale

Based on the construction of the BBC model with variable returns to scale and using DEAP2.1 software, the efficiency of the first and second stages of tourism poverty alleviation in the eight ethnic provinces and regions in 2003 and 2017 was calculated. The results are shown in Tables 1, 2.

**Table 1:** The efficiency of the first stage of poverty alleviation by tourism in the ethnic regions and its decomposition.

**Region**	**2003**				**2007**			
	**Overall** **efficiency**	**Pure technical** **efficiency**	**Scale** **efficiency**	**Return to** **scale**	**Overall** **efficiency**	**Pure technical** **efficiency**	**Scale** **efficiency**	**Return to** **scale**
Guangxi	1.000	1.000	1.000	–	1.000	1.000	1.000	–
Inner Mongolia	0.562	0.683	0.823	irs	0.915	0.939	0.975	irs
Guizhou	1.000	1.000	1.000	–	1.000	1.000	1.000	–
Yunnan	1.000	1.000	1.000	–	1.000	1.000	1.000	–
Tibet	1.000	1.000	1.000	–	0.590	1.000	0.590	irs
Qinghai	0.838	1.000	0.838	irs	1.000	1.000	1.000	–
Ningxia	0.370	0.584	0.633	irs	0.954	1.000	0.954	irs
Xinjiang	0.629	0.631	0.996	irs	1.000	1.000	1.000	–
Mean	0.800	0.862	0.911		0.932	0.992	0.940	

*irs, increasing returns to scale; –, constant returns to scale*.

**Table 2:** The efficiency of the second stage of poverty alleviation by tourism in the ethnic regions and its decomposition.

**Region**	**2003**				**2007**			
	**Overall** **efficiency**	**Pure technical** **efficiency**	**Scale** **efficiency**	**Return to** **scale**	**Overall** **efficiency**	**Pure technical** **efficiency**	**Scale** **efficiency**	**Return to** **scale**
Guangxi	0.119	1.000	0.119	drs	0.086	0.900	0.096	drs
Inner Mongolia	0.425	1.000	0.425	drs	0.343	1.000	0.343	drs
Guizhou	0.295	0.839	0.352	drs	0.098	0.875	0.112	drs
Yunnan	0.123	0.955	0.128	drs	0.057	0.915	0.062	drs
Tibet	1.000	1.000	1.000	–	1.000	1.000	1.000	–
Qinghai	1.000	1.000	1.000	–	0.846	0.999	0.847	drs
Ningxia	1.000	1.000	1.000	–	1.000	1.000	1.000	–
Xinjiang	0.293	1.000	0.293	drs	0.268	0.914	0.293	drs
Mean	0.532	0.974	0.540		0.462	0.951	0.469	

*irs, increasing returns to scale; –, constant returns to scale*.

In Table 1, it can be seen that the average combined efficiency of the first stage of the ethnic areas was 0.800 in 2003, and in 2017, this mean value rose to 0.932, achieving improvement. In terms of overall efficiency, four provinces reached DEA effectiveness in 2003, namely, Guangxi, Guizhou, Yunnan, and Tibet, increasing to five regions in 2017, namely, Guangxi, Guizhou, Yunnan, Qinghai, and Xinjiang. In addition, Qinghai had higher integrated efficiency in 2003, Inner Mongolia and Xinjiang had lower integrated efficiency due to lower pure technical efficiency, and Ningxia had lower pure technical efficiency and scale efficiency. The combined efficiency of Inner Mongolia, Ningxia and Xinjiang improved significantly in 2017, with Inner Mongolia and Xinjiang mainly due to the pull of pure technical efficiency, but scale efficiency did not improve much, while Ningxia improved both pure technical efficiency and scale efficiency. On the whole, the level of resource allocation and management in ethnic areas in the first stage has been improved, but the scale of resource input needs to be further optimized.

In Table 2, it can be seen that the average combined efficiency of the second stage in the ethnic areas is 0.532 in 2003 and 0.462 in 2017, which is much lower than the average combined efficiency of the first stage. It shows that although the investment in the tourism industry in ethnic areas has contributed to the development of the tourism industry, the development of the tourism industry has not been efficient in alleviating poverty in ethnic areas. From the perspective of DEA effectiveness, Tibet, Qinghai, and Ningxia provinces reached effectiveness in 2003, and only Tibet and Ningxia provinces reached DEA effectiveness in 2017. In terms of scale payoffs, provinces with decreasing scale payoffs predominated in both 2003 and 2017, suggesting that these provinces have a relative excess of inputs and underutilized resources. Overall, most regions had lower combined efficiency in 2003, mainly due to lower scale efficiencies. Pure technical efficiency and efficiency of scale declined in some provinces in 2017, resulting in a decline in the combined efficiency of both these areas. In addition, the pure technical efficiency of the two stages is higher, but compared with the first stage, the scale efficiency of the second stage is lower. It can be seen that the low scale efficiency is an important reason hindering the efficiency improvement of the second stage.

### Dynamic Efficiency Analysis Based on Malmquist Index

According to the statistics of poverty alleviation by tourism in ethnic areas from 2003 to 2017, the DEAP2.1 software is used to calculate and decompose the total factor productivity change index of the first and second stages, and further analyze the changes in the efficiency of the two stages, and the specific results of the operation are shown in Tables 3, 4.

**Table 3:** Malmquist index and its decomposition in the first stage from 2003 to 2017.

**Vintage**	**Technical efficiency** **change**	**Technological progress** **change**	**Pure technical** **efficiency change**	**Scale efficiency** **change**	**Total factor** **productivity change**
2003–2004	1.136	1.097	1.107	1.027	1.246
2004–2005	1.140	0.829	1.062	1.074	0.945
2005–2006	0.822	1.031	0.921	0.892	0.847
2006–2007	1.082	0.881	1.000	1.083	0.953
2007–2008	0.944	0.898	1.057	0.893	0.848
2008–2009	1.036	0.711	0.977	1.060	0.737
2009–2010	1.016	0.700	1.048	0.969	0.711
2010–2011	1.012	0.894	1.003	1.008	0.905
2011–2012	1.019	0.856	0.975	1.045	0.873
2012–2013	0.961	0.798	0.976	0.984	0.767
2013–2014	1.076	1.012	1.049	1.026	1.088
2014–2015	1.081	0.898	1.012	1.069	0.971
2015–2016	0.868	1.043	0.951	0.913	0.905
2016–2017	1.060	0.900	1.043	1.016	0.954
Mean	1.014	0.889	1.012	1.002	0.901

**Table 4:** Malmquist index and its decomposition in the second stage from 2003 to 2017.

**Vintage**	**Technical efficiency** **change**	**Technological progress** **change**	**Pure technical** **efficiency change**	**Scale efficiency** **change**	**Total factor** **productivity change**
2003–2004	0.965	0.954	0.992	0.973	0.921
2004–2005	0.903	1.024	1.031	0.876	0.925
2005–2006	1.116	0.866	0.991	1.127	0.967
2006–2007	1.056	1.041	0.992	1.065	1.099
2007–2008	0.801	1.673	0.990	0.808	1.339
2008–2009	1.203	0.810	1.006	1.196	0.975
2009–2010	1.024	1.184	0.993	1.031	1.212
2010–2011	0.998	1.015	1.012	0.986	1.012
2011–2012	1.146	0.896	0.996	1.150	1.027
2012–2013	0.771	1.308	1.014	0.761	1.009
2013–2014	0.982	0.915	0.973	1.009	0.898
2014–2015	0.978	0.924	0.991	0.987	0.903
2015–2016	1.078	0.950	1.000	1.078	1.024
2016–2017	0.774	1.204	0.996	0.778	0.932
Mean	0.976	1.035	0.998	0.978	1.011

**Figure 2:**
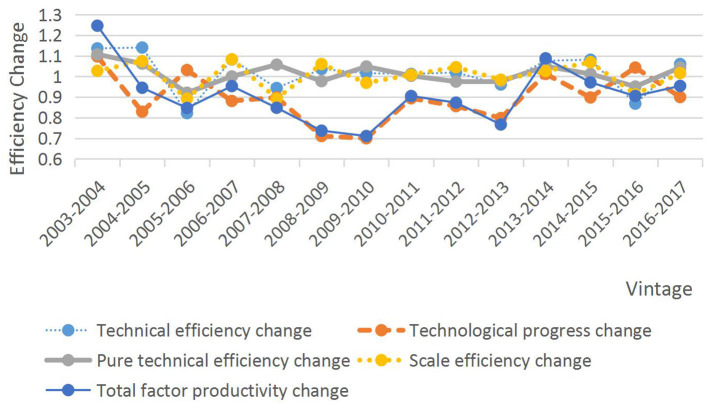
Malmquist index and its decomposition in the first stage from 2003 to 2017.

In Table 3 and Figure 2, it can be seen that the TFP index for the first stage of the 2003–2017 period is <1, except in 2003–2004 and 2013–2014 when it was >1. Its mean value is also 1, indicating a downward trend in overall TFP in the first stage over the past 15 years, with a rate of decline of 9.9%. Among them, the largest decline was in 2009–2010, reaching 28.9%, mainly due to the decline in the rate of technological progress. Further analysis shows that the mean value of the technical efficiency change index from 2003 to 2017 is 1.014, and the mean value of the technical progress index is 0.889, indicating that the fluctuation of the total factor productivity index is mainly affected by the fluctuation of the technical progress index, so the key to improving the level of total factor productivity in the first stage lies in the technological progress of the tourism industry. Since 2003, the average values of the pure technical efficiency change index and the scale efficiency change index have increased by 1.2 and 0.2%, respectively, but both have been declining for part of the period, indicating that the resource allocation and scale of tourism in ethnic areas also need further improvement in the first stage.

**Figure 3:**
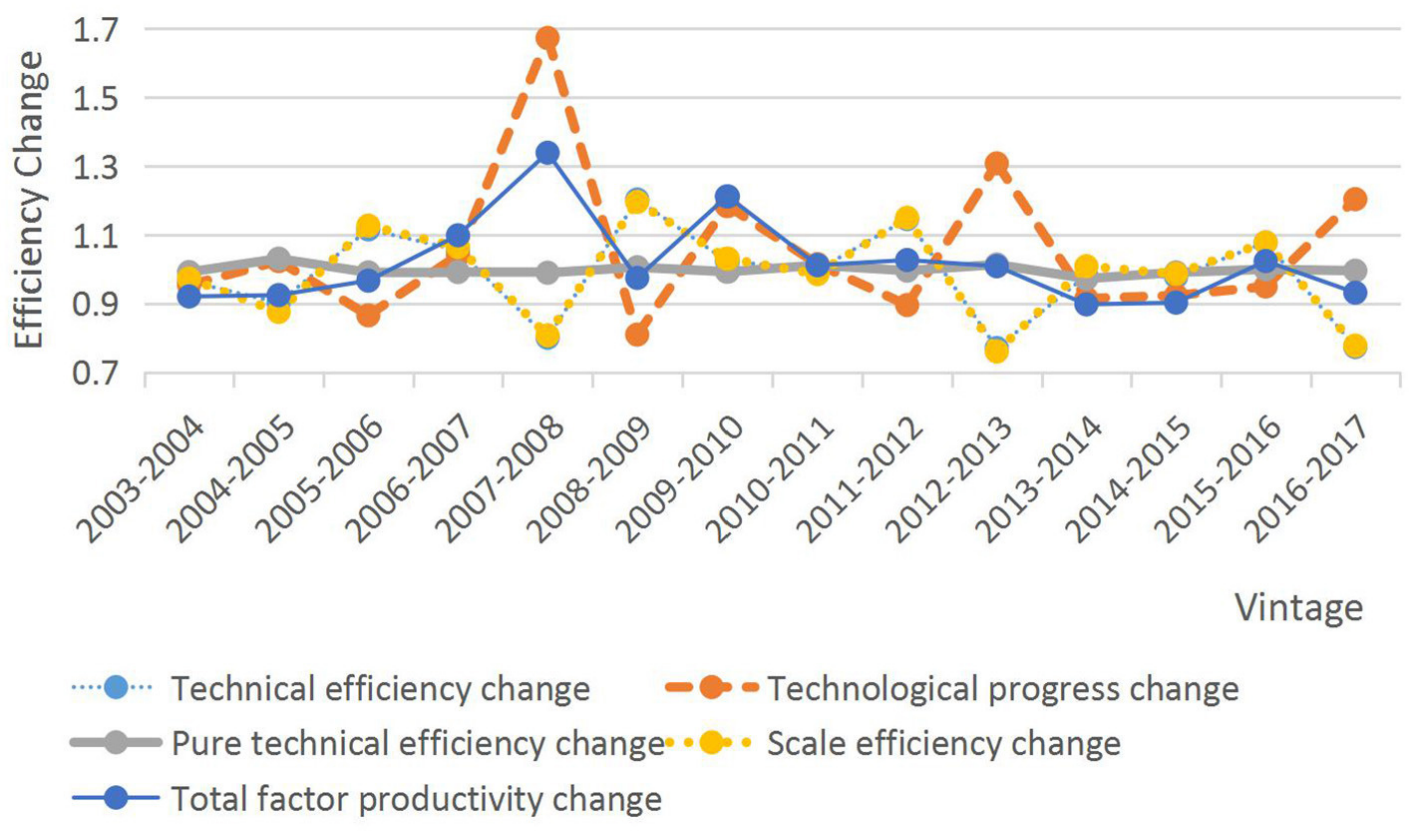
Malmquist index and its decomposition in the second stage from 2003 to 2017.

As can be seen in Table 4 and Figure 3, the average value of the total factor productivity (TFP) index for the second stage was 1.011 in 2003–2017, indicating that TFP grew at an average rate of 1.1% per year and that the increase was most pronounced in 2007–2008, mainly due to technological progress. Further analysis shows that the mean value of the technical efficiency change index is 0.976, the mean value of the technical progress index is 1.035, and the growth rate of technical progress in the six time periods is greater than the growth rate of technical efficiency, indicating that the improvement of total factor productivity mainly depends on technical progress. In addition, pure technical efficiency and scale efficiency generally showed a declining trend from 2003 to 2017, making the overall change in technical efficiency also declining, suggesting that the main reason hindering the growth of technical efficiency is that both pure technical efficiency and scale efficiency are low. In general, there is still a lot of room for improvement in the second stage of tourism management level and technical allocation, resource allocation, and scale. Tables 3, 4 show the results of the calculations of the Malmquist index and its decomposition in the time dimension for the two phases, and the changes in TFP and its decomposition for specific provincial regions in the two phases are shown in Tables 5, 6.

**Table 5:** Malmquist index and its decomposition in the first stage of ethnic regions.

**Region**	**Technical efficiency** **change**	**Technological progress** **change**	**Pure technical** **efficiency change**	**Scale efficiency** **change**	**Total factor** **productivity change**
Guangxi	1.000	0.908	1.000	1.000	0.908
Inner Mongolia	1.035	0.942	1.023	1.012	0.975
Guizhou	1.000	0.969	1.000	1.000	0.969
Yunnan	1.000	0.893	1.000	1.000	0.893
Tibet	0.963	0.849	1.000	0.963	0.818
Qinghai	1.013	0.864	1.000	1.013	0.875
Ningxia	1.070	0.833	1.039	1.030	0.892
Xinjiang	1.034	0.863	1.033	1.000	0.892
mean	1.014	0.889	1.012	1.002	0.901

**Table 6:** Malmquist index and its decomposition in the second stage of ethnic regions.

**Region**	**Technical efficiency** **change**	**Technological progress** **change**	**Pure technical** **efficiency change**	**Scale efficiency** **change**	**Total factor** **productivity change**
Guangxi	0.977	1.098	0.992	0.985	1.073
Inner Mongolia	0.985	1.074	1.000	0.985	1.058
Guizhou	0.924	1.113	1.003	0.921	1.029
Yunnan	0.947	1.066	0.997	0.949	1.009
Tibet	1.000	0.985	1.000	1.000	0.985
Qinghai	0.988	1.020	1.000	0.988	1.008
Ningxia	1.000	0.953	1.000	1.000	0.953
Xinjiang	0.994	0.984	0.994	1.000	0.978
Mean	0.976	1.035	0.998	0.978	1.011

As can be seen in Table 5, the annual average TFP indices for the first stage in the ethnic regions of China are all <1, indicating a downward trend in TFP in all provinces. Among them, the pure technical efficiency change index and scale efficiency change index in Inner Mongolia and Ningxia are both >1; the pure technical efficiency in Qinghai is unchanged, while the scale efficiency has improved; and the scale efficiency in Xinjiang is unchanged, while the pure technical efficiency change index is >1. At the same time, the technical efficiency change index of these four provinces is >1, but the technical progress index is <1, indicating that technological progress is the main factor affecting the changes in total factor productivity in these four regions. Although the pure technical efficiency and scale efficiency of Guangxi, Guizhou, and Yunnan remain unchanged, and their technical efficiency has not changed, the decline in technology has led to a decline in the total factor productivity of these four provinces. In addition, the low technological progress index is also the reason for the decline in Tibet's total factor productivity. On the whole, the total factor productivity of each region in the first stage was declining, mainly due to technological progress.

In Table 6, it can be seen that the second stage of TFP in ethnic areas basically shows an increasing trend, with Tibet, Ningxia, and Xinjiang showing declines of 1.5, 4.7, and 2.2%, respectively, mainly due to the decline in the rate of change of technological progress. In terms of technical efficiency, it declined in all six provinces except Tibet and Ningxia. In terms of technological progress, except for Tibet, Ningxia, and Xinjiang, there is an upward trend in technological progress in all regions. In terms of pure technical efficiency, only Guizhou has seen an increase, indicating that both the technical and management levels in ethnic regions have yet to be improved. In terms of scale efficiency, ethnic regions show a constant or declining trend, indicating that the level of resource allocation in ethnic regions also needs to be improved. Overall, although there were differences in the rate of change in TFP in the second stage, the differences were small, only Tibet, Ningxia, and Xinjiang were <1, and almost all of the remaining provinces achieved efficiency gains. The largest increase in TFP in Guangxi was 7.3%, indicating that Guangxi contributed the most to changes in TFP in ethnic regions. Compared with the first stage, the total factor productivity change rate of the provinces in the second stage has been significantly improved due to the increase in the change rate of technological progress.

### Analysis of the Efficiency Types of Tourism Poverty Alleviation

**Figure 4:**
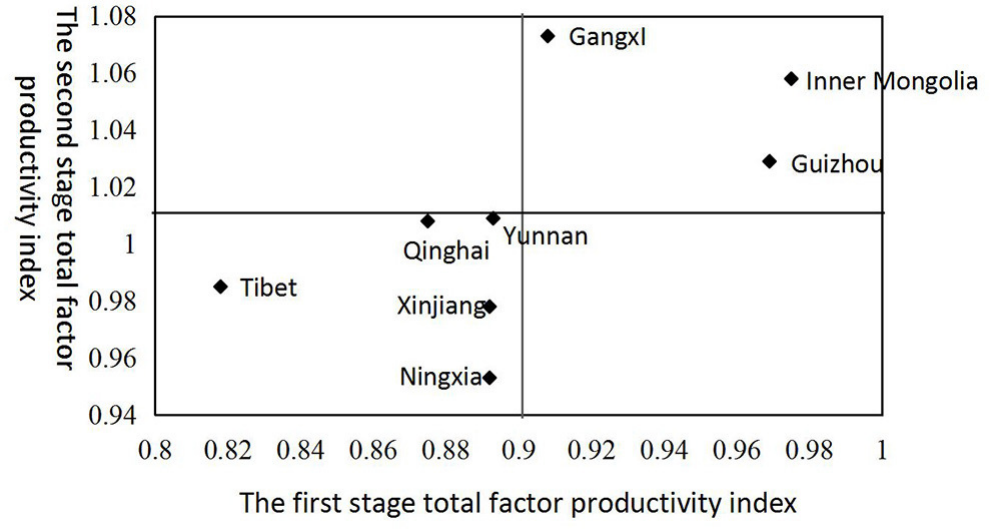
The distribution of efficiency in the first and second stages of the eight ethnic provinces.

In order to facilitate the comparative analysis of the empirical results, this paper further uses the Malmquist index values of the two stages, taking the average value of the Malmquist index in the first stage (0.901) and the average value of the Malmquist index in the second stage (1.011) as the mass point coordinates, and the total factor productivity index is the horizontal axis, and the second-stage total factor productivity index is the vertical axis; the efficiency level of tourism poverty alleviation in ethnic areas is divided into four categories, namely, “double-high” type, “low-high” type, “double-low” type, and “high-low” type. It is shown in Figure 4.

From the results of cluster classification, the total factor productivity index values of ethnic regions are concentrated in the “double high” and “double low” types. Among them, the “double high” type includes Guangxi, Inner Mongolia, and Guizhou, which are located in the upper right quadrant. Although the two-stage efficiency of these three regions is relatively high, the second-stage efficiency of these three provinces is about 1.053, while the efficiency of the first stage is only around 0.951, indicating that the three provinces should strive to improve the efficiency of the first stage while consolidating the efficiency of the second stage. The “double-low” type includes Qinghai, Yunnan, Tibet, Xinjiang, and Ningxia, which are located in the lower left quadrant. The two-stage efficiency of these five provinces is low. It shows that these five regions should first improve the efficiency of the first stage or the second stage, and then gradually develop to the stage with higher efficiency in both stages. It should be pointed out that the efficiency of the two stages is relative. Therefore, on the whole, the efficiency of the second stage in my country's ethnic regions is significantly higher than the efficiency of the first stage.

## Conclusions and Recommendations

### Conclusion

This paper establishes a two-stage evaluation model for the efficiency of tourism poverty alleviation and uses the DEA method to evaluate the efficiency of tourism poverty alleviation in China's ethnic regions from 2003 to 2017. From the above analysis, it can be seen that tourism poverty alleviation has brought about an improvement in the living standards of the residents in the ethnic regions of China and the development of the local economy, but the efficiency of tourism poverty alleviation needs to be improved. The results are as follows:

1. In the first stage, the level of resource allocation and management in the ethnic minority areas has improved considerably, and the pure technical efficiency is relatively high, but there is still a need to further optimize the allocation of tourism resources and the scale of investment in the ethnic minority areas; in the first stage, the total factor productivity of each region has shown a downward trend, and the key to enhancing it lies in promoting the technical progress of the tourism industry.
2. Pure technical efficiency is also high in the second stage, but scale efficiency is low, which hinders the efficiency improvement in this stage, and should focus on the management level and technical allocation of tourism, resource allocation, and scale. In the second stage, the change rate of total factor productivity in the provinces has increased significantly, and the change rate of technological progress in the tourism industry should continue to be increased.
3. The “double-high” type includes Guangxi, Inner Mongolia, and Guizhou, indicating that these provinces should focus on improving the efficiency of the first stage while stabilizing the efficiency of the second stage. The “double-low” model includes Qinghai, Yunnan, Tibet, Xinjiang, and Ningxia, implying that these five regions should increase investment in the tourism industry, promote the development of the tourism industry, and focus on the benefits of the poor in the process of tourism development.

### Recommendations

Based on the aforementioned two-stage analysis of the efficiency of tourism poverty alleviation, China has demonstrated in practice that developing tourism is one of the effective ways to help poor areas escape poverty and become rich. Tourism has the characteristics of strong pull, poverty alleviation, and benefiting a wide range of people, making it quickly become a way to alleviate poverty in several areas. Through tourism poverty alleviation, great changes have taken place in ethnic regions. Due to the different development of resources in different regions, it is necessary to take targeted measures based on the actual conditions of each province. This paper puts forward the following suggestions:

First, broaden the financing channels for tourism poverty alleviation. In addition to relying on government investment and national bank loans for funding tourism poverty alleviation, local commercial banks should appropriately expand the scale of credit for some tourism poverty alleviation to nurture the development of tourism poverty alleviation in poor areas. At the same time, ethnic regions should seize the opportunity of the country's relevant policies on poverty alleviation and actively guide investment in tourism for poverty alleviation through measures such as upward struggle, external introduction, and internal revitalization. In addition, social capital is encouraged to participate in tourism poverty alleviation fairly, and enterprises of various ownerships are encouraged to invest in tourism poverty alleviation in ethnic areas according to law. Broaden domestic and foreign financing channels, use financial funds, private capital and foreign capital, broaden diversified funding sources, cultivate and develop a group of private, foreign, and mixed economic structure investment and financing entities, improve the efficiency of capital use, and help poverty alleviation in ethnic areas raise more funds. Specifically, the government can guide social capital to increase investment in tourism poverty alleviation through financial subsidies and loan interest discounts, and guide diversified funds such as private capital and industrial funds to support tourism poverty alleviation by supporting tourism enterprises to integrate and develop with related industries through the exchange of property rights and cooperative development, and encourage private capital to intervene in government-led poverty alleviation supporting infrastructure construction through BT (build-transfer), BOT (build–operate–transfer), and other methods (Yang and Shi, 2014). Attract domestic or regional enterprises with certain economic strength, good reputation, and business expansion intention to develop tourism projects in the local area. In addition, part of the national poverty alleviation funds and related enterprises with good local efficiency and support for poverty alleviation will be used to establish a tourism development poverty alleviation fund, giving priority to those poor counties that have the ability to implement the tourism poverty alleviation model and promote the development of tourism. By increasing investment in tourism, the tourism industry can play its role as a “growth pole” and drive the ethnically impoverished areas to break through the “poverty vicious circle” proposed by Nacks and jump out of the “low-level equilibrium trap” proposed by Nelson.

Second, improve pure technology and scale efficiency. Technological progress is an extremely important way to promote the efficiency of tourism poverty alleviation. Through the above empirical research results, it can be seen that the overall efficiency is affected and restricted by pure technical efficiency and scale efficiency, and their effects on tourism input and tourism output stages are different. Therefore, at the stage of investment in the tourism industry, attention should be paid in improving the pure technical efficiency of tourism poverty alleviation in various provinces. Local governments should create a favorable investment environment for investors, actively introduce various types of investment, rationally allocate tourism resources, explore the “big data + tourism” poverty alleviation development model, and improve the scientific and technological level of tourism development, so as to realize the effective utilization of economic factors, thereby enhancing the comprehensive efficiency of tourism poverty alleviation. Tourism poverty alleviation in ethnic areas needs to seize the opportunity of the central government's implementation of the digital rural strategy, make full use of the advantages of new ideas, new technologies, and new industries, combine with poverty alleviation work, boldly explore and innovate the “big data + tourism” poverty alleviation mechanism, relying on the e-commerce platform to promote the sales of tourism products and agricultural and sideline products in ethnic areas. Accelerate the construction of a comprehensive service platform for smart tourism, and at the same time carry out informatization and informatization skill poverty alleviation for the poor. Focus on integrating the power of informatization construction such as “Village to Village” and “Distance Education,” extensively utilize the advantages of big data information resources, organize network poverty alleviation training activities, and set up virtual forums for big data + tourism poverty alleviation to improve the quality of the poor, thereby improving the efficiency of tourism poverty alleviation. At the stage of tourism output, it is necessary to scientifically judge the best scale of local tourism development, actively build a complete tourism industry chain, develop distinctive tourism industry brands, and continuously improve the scale benefits of tourism development.

Third, optimize the industrial structure and protect the environment. Optimizing the industrial structure is conducive to enhancing industrial competitiveness and giving play to regional advantages. Ethnic regions should rely on their own unique resource advantages and foundations, such as unique natural landscape and climate, distinctive regional food culture, strong national culture, and unique tourism products of ethnic minorities, give full play to the characteristics of the tourism industry's high degree of relevance, low employment threshold, strong comprehensive driving force, and large radiation traction, etc., to drive regional economic growth and employment of impoverished residents. In the ever-changing market demand, giving full play to the resource allocation and regulation role of the market economy and actively promoting the integrated development of the tourism industry and other industries, especially emerging industries, such as digital information and new energy, introduce energy-saving and environmentally friendly industrial forms, and actively eliminate outdated production capacity with high-energy consumption. In the process of developing tourism for poverty alleviation in ethnic areas, they should also actively cooperate with scientific research institutions and universities to improve the level of science and technology, actively carry out environmental governance and maintenance, establish a good environment and basic conditions for the development of the tourism industry, and ensure the sustainable development of work of regional economy and tourism poverty alleviation (Li, 2018).

Finally, play the synergy of multiple subjects. Government departments should play a leading role and supervisory function in the process of tourism poverty alleviation, make reasonable arrangements for poverty alleviation development in ethnic regions, and overall planning for economic development. The government should carry out tourism-related skill training in accordance with the actual conditions in ethnic areas to improve the overall quality of local residents' learning ability, service level, professional skills and management ability, and enhance their ability to work. Poverty alleviation is one of the social responsibilities that enterprises and social organizations should actively fulfill. Enterprises can help expand the sales channels of tourism products in ethnic areas based on their own advantages in technology, resources, and channels, build an information sharing platform, help promote the image of tourist destinations and enhance the popularity of local tourism, and at the same time provide training in job skills and employment opportunities for impoverished residents, helping to solve the problems of surplus labor and difficulties in starting up businesses for migrant workers. Poor residents are an important force in the fight against poverty. Considering the sustainable development of tourism poverty alleviation in ethnic areas, local residents should be encouraged to rely on their handicraft skills, land, housing, and other resources to engage in employment activities such as tourism services, the production of special handicraft products, the sale of tourism souvenirs, and the establishment of farmhouse homes, coordinating the relationship between the development of tourism poverty alleviation and the benefits to residents, and promoting their participation in tourism poverty alleviation, so that residents can lift themselves out of poverty and improve their living standards while raising the level of local economic development. Considering the sustainable development of tourism and poverty alleviation in poor areas, local residents should be encouraged to rely on their handicraft skills, land, houses, and other resources to engage in employment activities such as tourism services, production of special handicraft products, sale of tourist souvenirs, and establishment of farmhouses. In addition, the relationship between the development of tourism poverty alleviation and the benefit of poor residents should be coordinated to promote the participation of poor residents in poverty alleviation, so that poor residents can get out of poverty and improve their living standards while increasing the level of local economic development.

### Research Gaps and Outlook

Due to data limitations, the research cycle of this paper is relatively short. In the future, as the data continue to improve, relevant studies may add tests for the generalizability of the conclusions in this paper to obtain more reliable conclusions. In addition, the selection of evaluation indicators was influenced by the availability of data and did not fully take into account the impact of indicators such as health, education, social welfare, etc., and more evaluation indicators could be added in the future to improve the accuracy of the results.

## Data Availability Statement

The raw data supporting the conclusions of this article will be made available by the authors, without undue reservation.

## Ethics Statement

The studies involving human participants were reviewed and approved by the Academic Committee of Guizhou University of Finance and Economics. Written informed consent to participate in this study was provided by the participants' legal guardian/next of kin.

## Author Contributions

JY conceived the study and wrote the manuscript. YW wrote and revised the manuscript. JW analyzed the data. CW conceived the study and revised the manuscript. QW revised the manuscript. All authors contributed to the article and approved the submitted version.

## Conflict of Interest

The authors declare that the research was conducted in the absence of any commercial or financial relationships that could be construed as a potential conflict of interest.

## Footnotes

^1^The distance function is a tool for studying multi-input and multi-output technical systems without making any assumptions about the behavior of producers.
